# Quality of life and impact of pain in women treated with aromatase inhibitors for breast cancer. A multicenter cohort study

**DOI:** 10.1371/journal.pone.0187165

**Published:** 2017-11-08

**Authors:** Françoise Laroche, Serge Perrot, Terkia Medkour, Paul-Henri Cottu, Jean-Yves Pierga, Jean-Pierre Lotz, Karine Beerblock, Christophe Tournigand, Laure Chauvenet, Didier Bouhassira, Joël Coste

**Affiliations:** 1 Pain Clinic, Saint Antoine Hospital, Paris, France; 2 INSERM U 987 and U 938, Paris, France; 3 Pain Clinic and Internal Medicine Department, Hôtel Dieu Hospital, Paris, France; 4 Medical Oncology Department, Institut Curie, Paris, France; 5 Oncology Department, Tenon Hospital, Paris, France; 6 Oncology Department, Mondor Hospital, Créteil, France; 7 Oncology Department, Hôtel Dieu Hospital, Paris, France; 8 Biostatistics and Epidemiology Unit, Hôtel Dieu Hospital, Paris, France; Tokai Daigaku, JAPAN

## Abstract

Women with hormone-dependent breast cancer are treated with aromatase inhibitors (AI) to slow disease progression by decreasing estrogen levels. However, AI have adverse effects, including pain, with potentially serious impact on quality of life (QOL) and treatment compliance. We evaluated quality of life during the first year of AI treatment, focusing particularly on the impact of pain. In a multicenter cohort study of 135 women with early-stage breast cancer, free of pain at the initiation of AI treatment, quality of life (by the EORTC QLQ-BR23), somatic and psychic symptoms, psychological characters, temperament and coping strategies were assessed at baseline and at each follow-up visit (1, 3, 6 and 12 months). The impact of treatment-induced pain on quality of life during follow-up was determined with repeated-measures regression models. These models were constructed to assess the effects of pain and pain type on quality of life during follow-up, taking into account predictors associated with quality of life at baseline. Prior ganglion resection, taxane treatment and chemotherapy, a high amplification score on the pain catastrophizing scale, and a high harm avoidance score on the personality questionnaire were associated with a significantly lower baseline QOL. Fifty-seven percent of women developed pain of five different types: upper or lower limb joint pain, diffuse pain, neuropathic pain, tendon pain and mixed pain. A significant decrease in QOL was noted in the women with pain, particularly for body image, sexual functioning and future perspectives. Moreover, the impact of pain on QOL depended on the type of pain experienced. In conclusion, women treated with aromatase inhibitors display changes in quality of life and the degree of change in quality of life depends mostly on the type of pain experienced. Oncologists and patients should be aware of painful adverse effects of AI and encouraged to provide or receive earlier and more appropriate management of these effects.

## Introduction

Estrogen deprivation therapy with aromatase inhibitors (AIs) is now the first-line treatment for hormone-dependent breast cancers, and is prescribed for up to 60% of patients. Aromatase inhibitor-related pain remains a key issue, mostly during the first year of treatment [1, 2].

The quality of life (QOL) of patients with breast cancer is worsened by the cancer itself, but also by treatment of the disease. Treatment impact depends on the treatment administered, with differences between types of chemotherapy and types of surgery (i.e. mastectomy vs. tumorectomy) [3, 4]. Simple biopsy and ganglion resection also have significant impacts on quality of life, mostly due to anxiety [5]. Quality of life is also decreased by the cognitive consequences of treatment [6]. However, clinical trials and prospective surveys have yielded conflicting results concerning quality of life, with some reporting an improvement over time, and others a degradation, even after three years of follow-up [7]. Joint and muscle pain decreases quality of life in some breast cancer patients [8]. Indeed, a significant association has been found between musculoskeletal impairment, depression and fatigue in breast cancer survivors within the first year after treatment [9].

Clinical trials and prospective studies have indicated that up to 50% of patients develop pain following the initiation of AI treatment [10, 11, 12, 13]. However, few studies have assessed the impact of AIs on QOL in treated women, particularly for those experiencing pain. We described the various types of musculoskeletal pain experienced by women on AI treatment in a previous study [14]. In total, 57% (77 of 135) of the women in this study developed pain of five types: joint pain 48 (36%), widespread pain 30 (22%), neuropathic pain in the legs 12 (9%), tendonitis 29 (22%) and mixed pain (27%), beginning, on average, three to six months after treatment initiation. The pain was severe enough for AI treatment to be stopped in 12 patients. We identified risk factors for pain which were psychological or related to quality of life. Genetic factors, inflammation, immune and hormonal status had no effect on induced AIs pain.

The aims of the present study were to evaluate QOL in women treated with AIs and to comprehensively assess the impact of pain and type of pain on QOL during the first year of AI treatment.

## Patients and methods

### Design

We carried out a one-year observational multicenter prospective cohort study, at four medical oncology departments and one pain clinic located in different university hospitals in Paris, France.

### Ethics statement

This study was carried out in accordance with the Helsinki Declaration concerning the use of human subjects in biomedical research. Written informed consent was obtained from each subject before enrollment at inclusion visit, before starting AI treatment (from June 2009 to March 2011). Approval was obtained from the institutional review board and the French data protection agency (CCTIRS, CNIL) before subject enrollment and data collection.

### Study population

Consecutive women treated for early breast cancer at four medical oncology departments were eligible to participate in this study if they were starting AI treatment and had no pain at treatment initiation. Informed consent was obtained from all participants, who were then referred to the same pain clinic for an initial visit, which included the completion of study questionnaires and blood sampling. The enrollment period lasted 16 months, from June 2009 to March 2011, and each participant was followed for 12 months. The exclusion criteria were: other pain conditions interfering with pain assessment, no written consent, patient not able to follow the protocol, other treatment for cancer scheduled for the next 12 months, previous AI treatment, and estimated survival of less than 12 months.

### Aromatase inhibitor treatments and other drugs prescribed during the study period

All postmenopausal women with hormone-dependent breast cancer recruited for this study began oral treatment with AI, prescribed by their oncologist, as an adjuvant therapy to be continued for at least five years, after curative cancer treatment. Patients were prescribed non-steroidal (1 mg anastrozole, 2.5 mg letrozole) or steroidal (25 mg exemestane) AIs, to be taken daily. Patients considered at risk of osteoporosis were also prescribed anti-osteoporotic treatments, principally a combination of bisphosphonates and vitamin D supplements.

All other treatments, including analgesics and nonsteroidal anti-inflammatory drugs (NSAIDs), were permitted, and their use was monitored for the analyses. Analgesics were classified according to the WHO scale.

### Measurements

Baseline and follow-up measurements were carried out under the supervision of a research nurse and two rheumatologists specializing in pain medicine. Outcome measures were assessed at 1, 3, 6 and 12 months after the start of AI treatment. At each visit, a complete medical examination was carried out by a rheumatologist specializing in pain medicine and patients were asked to complete questionnaires concerning quality of life (EORTC QLQ-BR23), pain (VAS), psychological variables (HADS, PCS) and fatigue (MFI20).

#### Demographic and cancer history data

The demographic data collected included age, body mass index (BMI), menopause duration and history of hormonal replacement therapy. Cancer history variables included pathological subtype of breast cancer, type of surgery for breast cancer, history of chemotherapy and radiotherapy.

#### Pain and fatigue assessment

Current (average over the last seven days) and worst (in the last seven days) pain intensities were assessed with a 100-mm visual analog scale (VAS), with the endpoints “No pain” and “Worst possible pain” [15]. We considered pain as clinically significant for VAS scores of at least 30 mm. The multidimensional fatigue inventory (MFI-20) is a 20-item self-report instrument designed to measure fatigue through the following dimensions: general fatigue, physical fatigue, reduced activity, reduced motivation, and mental fatigue [16, 17].

#### Pain classification

As in a previous study [14], four classes of pain were considered: joint pain, diffuse pain, neuropathic pain, and tendon pain. Briefly, these classes were determined in several steps, using: 1) the body diagram included in the Brief Pain Inventory [18–20] for pain localization and classification of diffuse pain; 2) first 7 items from the DN4 questionnaire [21] to detect neuropathic component; 3) specific questions on pain location (around the joint, inside the joint, etc.).

#### Psychological assessment

Anxiety and depression were assessed with the Hospital Anxiety and Depression scale (HADS) [22, 23], which is widely used and has been shown to be both reliable and valid. The HADS comprises seven questions relating to depression and seven to anxiety, and the questions are divided into two subscales allowing for detecting states of depression and anxiety. Personality traits and pain catastrophizing were assessed with the Temperament and Character Inventory (TCI-R French version) [24] and the Pain Catastrophizing Scale [25]. The Temperament and Character Inventory (TCI-R) is a 240-item questionnaire assessing seven dimensions of personality (novelty seeking, harm avoidance, reward recompense, persistence, self-directedness, cooperativeness, transcendence). The Pain Catastrophizing Scale (PCS) is a widely used measure of catastrophic thinking related to pain. It has been translated into several languages including French [26]. The PCS is a 13-item instrument evaluating four dimensions rumination, magnification and helplessness.

#### Determinations of hormone levels

Biological tests included circulating sex hormone concentrations (estradiol, estrone, delta 4 androstenedione, testosterone, sex hormone-binding protein) tested by radio-immunology Assay (RIA) with immunofluorescence.

#### Quality of life

Breast cancer-related quality of life was assessed with the EORTC breast cancer module (EORTC QLQ-BR23) [27]. This 23-item questionnaire yields scores for four functional scales (body image, sexual functioning, sexual enjoyment, and future perspective) and four symptom scales (arm symptoms, breast symptoms, side effects of systemic therapy, and being upset by hair loss) that are linearly transformed onto a 100-point scale. Higher scores for functioning indicate better functioning, and higher scores for symptoms indicate worse symptoms. No global score is available. This questionnaire has been shown to have excellent reliability and validity in breast cancer patients.

### Statistical methods

Standard univariate (means, and standard deviations) and bivariate (Wilcoxon tests) statistics were used to summarize and compare quality-of-life outcome variables (QLQ-BR23 scales, HAD scales).

Predictive models of quality of life scale scores were constructed in several stages. We first constructed a multiple regression model for predicting baseline scores, by entering variables into the model according to their temporal relationship to quality of life: 1) genetic variables, 2) information about cancer diagnosis and treatment, 3) clinical, psychological and biological variables measured at baseline, 4) the type of AI (steroidal or non-steroidal), and, finally, interactions between significant predictors. Regression coefficients and 95% confidence intervals (CI) for final multiple regression models are presented.

We then constructed mixed effects, repeated measures regression models to assess the effects of pain and pain type on quality of life during follow-up independently of, or taking into account, predictors associated with quality of life at baseline, and coping assessed at each follow-up visit (1, 3, 6 and 12 months). Multiple imputation techniques were used to derive estimates of missing scores at these time points, particularly for patients stopping anti-aromatase treatment due to pain (informative drop-outs). SAS version 9.2 (SAS Institute, Cary, North Carolina) was used for statistical analysis. All statistical tests were two-tailed, and values of *p* less than 0.05 were considered significant.

## Results

### Characteristics of the participants

**Fig 1:**
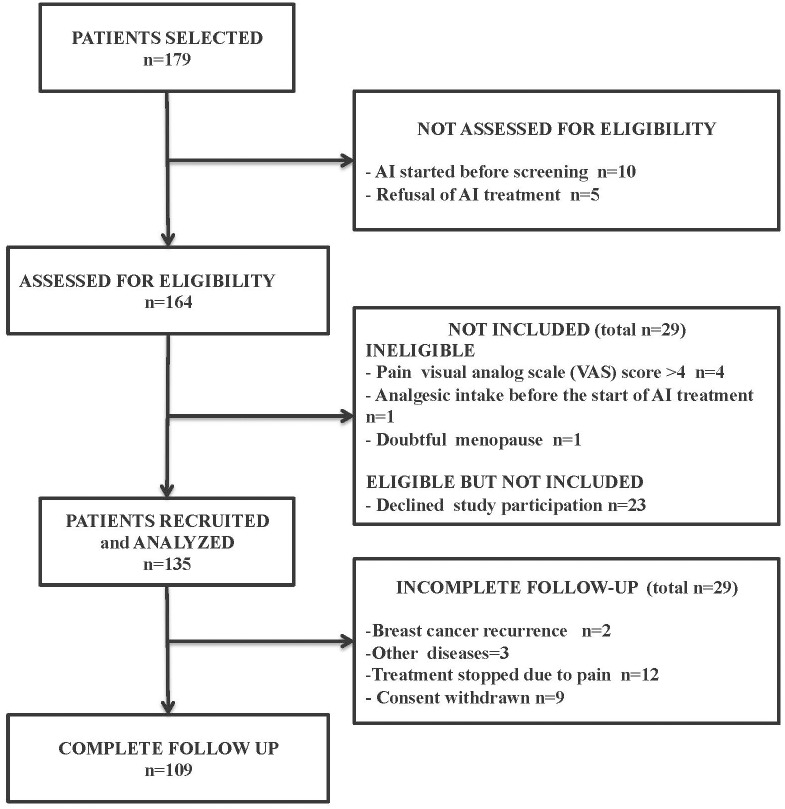
Flow Chart of the study.

One hundred and seventy-nine women with breast cancer but no pain were invited to participate in this study, just before they began AI treatment. In total, 44 of these women either declined participation or were excluded because they were not eligible. This left 135 women, who were recruited and for whom we obtained data. The demographic data are summarized in Table 1 (Table 1). All patients were Caucasian. The included women had a mean age of 61.5 (±7.1) years, and a mean BMI of 25.3±5.3 kg/m^2^. After recruitment, nine women withdrew consent, and 17 ended treatment early (2 due to breast cancer recurrence, 3 due to incidental diseases and 12 because of pain) (Fig 1). Letrozole was prescribed in 67%, anastrozole in 32% and exemestane in 1% of cases.

**Table 1:** Baseline characteristics of all patients.

Age (yrs)	61.5±7.1
BMI	25.3 ±5.3
Quality of life (EORTC QLQ-BR23)	
Body image	66.0±33.0
Sexual function	20.0 ± 22.6
Sexual enjoyment	52.0±3.5
Future perspectives	55.3±31.1
Systemic therapy side effects	32.0 ± 21.6
Breast symptoms	30.0 ± 23.0
Arm symptoms	24.2 ± 24.7
Upset by hair loss	57.3 ± 42.1
Psychological Factors	
Anxiety (HADs)	7.2 ± 3.8
Depression (HADs)	3.6±3.0
Personality traits (TCI)	
Novelty seeking (NS)	8.0 ± 2.5
Harm avoidance (HA)	8.6±4.6
Reward recompense (RD)	10.1±2.3
Persistence (P)	3.3 ± 1.4
Self-directedness (SD)	19.2 ± 3.8
Cooperativeness (C)	20.4 ± 2.8
Transcendence (ST)	6.3± 3.4
Pain catastrophizing (PCS)	14.3 ± 11.0
Rumination	5.9 ±4.4
Amplification	2.9 ± 2.6
Dispair	5.5 ± 4.8
Sleep, Not restorative (%)	28
Fatigue (MFI20)	59.7 ± 6.8
Fatigue—general	12.2±1.7
Fatigue—physical	11.0±4.1
Fatigue—mental	8.7±3.8
Fatigue—reduced activities	10.3± 3.8
Fatigue—motivation	8.6±3.9

Values are expressed as mean ± SD where otherwise indicated.

Pain developed during follow-up in 57% of the patients studied (*n* = 77; (Kaplan-Meier estimate of pain occurrence at 1 year: of 0.59, 95% confidence interval: 0.51–0.68). AI treatment was stopped because of pain in 12 cases. Pain incidence and a comparison of the characteristics of the women who developed pain and those of the women who remained pain-free have been described elsewhere [14]. We identified five primary types of pain: joint pain in 36%, diffuse pain in 22%, tendinitis in 22%, neuropathic pain in 9% and mixed pain in 11% of the women.

During the study period, patients with pain were prescribed various analgesics and non-steroidal anti-inflammatory drugs (NSAIDs): acetaminophen was prescribed to 48 women, oral NSAIDs to nine, topical NSAIDs to seven, and weak opioids (e.g., tramadol, codeine) to 24.

### QOL at baseline and during the first year of AI treatment

**Fig 2:**
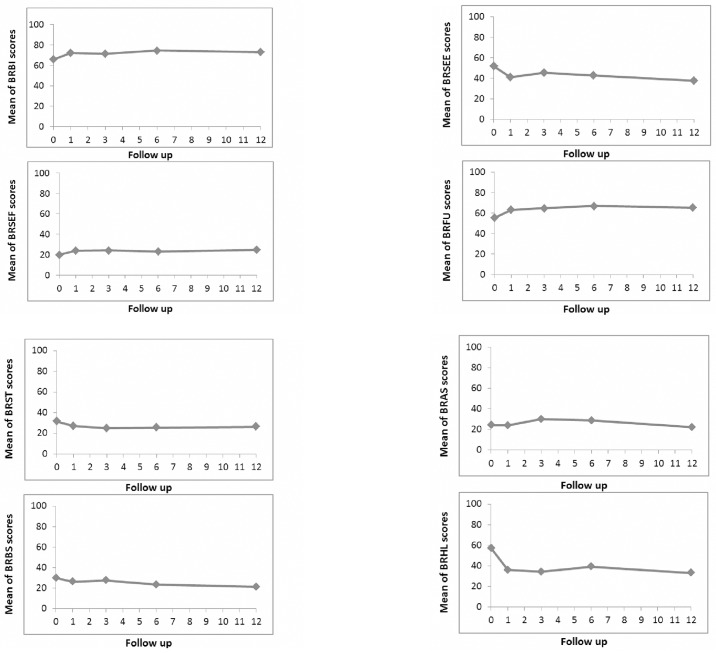
Quality of life follow up during the 12 months of AI treatment (EORTC QLQ-BR23 questionnaire). Mean scores of scales are shown: Body image (BRBI), Sexual functioning (BRSEF), Sexual enjoyment (BRSEE), Future perspective (BRFU); Systemic therapy side effects (BRST), Breast symptoms (BRBS), Arm symptoms (BRAS), Upset by hair loss (BRHL).

We present only six of the eight quality of life domains (3 functional and 3 symptoms) of the EORTC QLQ-BR23, because information about sexual enjoyment was not available for many women due to a lack of sexual activity, and the vast majority of the women had no hair loss. Mean QOL scores at baseline and during the first year of treatment are shown in Fig 2. During the 12 months of treatment, scores on QOL functional scales (especially body image and future perspective) remained relatively stable, except for a slight increase during the first month (of 8 and 9%, respectively). Scores for QOL symptom scales decreased slightly (by about 9%) during the first month and then remained stable.

### QOL predictors at baseline

A high magnification score on the pain catastrophizing scale (PCS) at baseline was associated with poor scores for several QOL dimensions, including sexual functioning, systemic therapy side effects, depression and anxiety (Table 2). A high harm avoidance score for the TCI questionnaire (TCI-HA) was associated with impairment in QOL domains. Indeed, each one-point increase in TCI-HA score was associated with a 2.33-point decrease in body image score, a 2.78-point decrease in future perspective score and slight increases in depression (0.18 point) and anxiety scores (0.43 point).

**Table 2:** Independent predictive factors for baseline quality of life* (BR-23 and HAD scales).

	Independent factor	Regression coefficient (95% confidence interval)	p
Body image (+)	Taxanes	-13,60 (-24.03 to -3.17)	0.01
	TCI-HA	-2,33 (-3.49 to -1.18)	<0.0001
Sexual Functioning (+)	Menopause duration	-0.56 (-1.09 to -0,02)	0.04
	PCS Rumination	-1.52 (-2.97 to -0.07)	0.04
	PCS Amplification	3.23 (0.76 to 5.71)	0.01
Future perspective (+)	TCI-HA	-2.78 (-3.84 to -1.72)	<0.0001
Systemic therapy side effects (-)	Chemotherapy	17.93 (10,78 to 25.10)	<0.001
	PCS Amplification	1.69 (0.39 to 3.00)	0.01
Breast symptoms (-)	BMI	0.74 (0.04 to 1.44)	0.04
	Taxanes	9.47 (2.07 to 16.88)	0.01
	Previous pain	16.41 (7.31 to 25,51)	0.0006
	Delta 4	-7.32 (-12.99 to -1.66)	0.012
Arm symptoms (-)	Ganglion resection	17.76 (5.71 to 29.83)	0.005
	SBP	-0.153 (-0.29 to -0.01)	0.04
HADS depression (-)	TCI HA	0.18 (0.07 to 0.29)	0.002
	TCI SD	-0.17 (-0.31 to -0.04)	0.01
	PCS amplification	0.21 (0.02 to 0.41)	0.03
HADS anxiety (-)	TCI HA	0.43 (0.31 to 0.54)	<0.0001
	TCI P	0.83 (0.46 to 1.19)	<0.0001
	PCS Amplification	0.32 (0.12 to 0.52)	0.002

* Final linear mixed models after imputation of missing values. Only regression coefficients with p-values < 0.05 are shown.

(+) refers to: function increase.

(-) refers to: symptom decrease.

Prior taxane treatment was associated with a 13.60-point decrease in body image score and an increase in breast symptom score of up to 9.47. Prior chemotherapy was associated with an increase in systemic side effects score of almost 18 points. We also confirmed that ganglion resection was associated with an increase in arm symptom score (of 18 points in our study).

Changes in hormone levels were associated with only two quality of life domains: a decrease in delta 4 androstenedione levels was associated with a significant increase in breast symptoms score (7.32 points) and a decrease in SBP levels was associated with an increase in arm symptoms score.

### Impact of pain type on QOL during follow-up

After adjustment on baseline predictors for each EORTC BR-23 scale, five different types of pain (upper or lower limb joint pain, diffuse pain, neuropathic pain, tendon pain and mixed pain) had significant but different effects on QOL (Table 3). Joint pain was associated with a lower body image score (-6.07), neuropathic pain was associated with a lower future perspective score (-21.57) and joint and diffuse pain were associated with higher systemic therapy side effect scores (5.76 and 7.14, respectively). All pain types were associated with higher arm symptom scores.

**Table 3:** Impact of pain on quality of life (BR-23 and HAD scales) during follow up: Regression coefficients* and 95% confidence interval.

	No pain (reference)	Joint pain (upper or lower limb)	Diffuse pain	Neuropathic pain (alone or mixed)	Tendinous pain	Mixed pain (other)	Adjustment covariates
Body Image (+)	0	-6.07 (-11.59 to -0.56)	-	-	-	-	Baseline body image, PCS Amplification,
Sexual Functionning (+)	0	-	-	-	-	-	
Future perspective(+)	0	-	-	-21.57 (-34.07 to -9.07)	-	-	Baseline future perspective, PCS Rumination, PCS Amplification, TCI HA
Systemic therapy side effects (-)	0	5.76 (1.91 to 9.62)	7.14 (1.88 to 12.41)	-	-	-	Baseline systemic therapy side effects, PCS Amplification, Baseline body mass index
Breast symptoms (-)	0	-	-	-	-	-	
Arm symptoms (-)	0	10.57 (4.70 to 16.43)	20.25 (12.56 to 27.94)	12.62 (1.51 to 23.72)	14.27 (5.35 to 23.19)	21.09 (13.67 to 28.50)	Baseline arm symptoms, PCS Amplification,
HADS depression (-)	0	-	-	-	-	-	
HADS anxiety (-)	0	-	-	-	-	-	

* Final linear mixed models after imputation of missing values. Only regression coefficients with p-values < 0.05 are shown.

(+) refers to: function increase.

(-) refers to: symptom decrease.

None of the types of pain had an impact on anxiety and depression (HADs scale) during the year of follow-up.

## Discussion

This prospective multicenter study using the EORTC QLQ-BR23, a specific QOL questionnaire for breast cancer, identified a number of factors predictive of baseline QOL of women treated with AI (before the start of AI treatment) and especially revealed that QOL of these women is dependent on the type of pain developed during the first year of AI treatment.

### QOL at baseline and during the first year of AI treatment

This study found that QOL function and symptom scores remained relatively stable over the first year of AI treatment whereas Kulesza-Bronczyk et al. reported an increase in QOL during the year following mastectomy in 110 women [28]. They concluded that symptoms resolve over time, leading to an increase in QOL. However, it was not specified whether the women in their study received AI shortly after surgery. The stable QOL scores over time in this study may result from the combination of a decrease in postsurgical symptoms and an increase in pain or discomfort due to AI treatment [14].

### Baseline QOL predictors

This study found that there was a relationship between breast cancer QOL score at baseline and a number of predictors, including having received prior chemotherapy, taxane treatment and ganglion resection. Similar relationships have been reported in various previous studies, including those by Miegg et al. and Sestak et al. [1, 29]. This study also found that baseline QOL was associated with a number of other factors, including catastrophizing, personality traits, anxiety and depression and revealed the magnitude of the effect of each predictor on baseline QOL scores.

Catastrophizing magnification and harm avoidance were independently associated with a poor QOL score at baseline. These two dimensions were implicated in more than three domains of QOL (including body image and future perspective) and were also associated with depression and anxiety scores. In patients with acute or chronic pain, catastrophizing has been shown to be a robust predictor of adverse pain outcomes. High levels of catastrophizing are associated with higher pain intensity, psychological distress and disability [30, 31].

The other baseline clinical variables considered, such as pain intensity (note that patients reporting pain with a VAS score > 4 at baseline were excluded), fatigue, sleep disorders, and hot flushes, were not associated with low baseline QOL scores in this study.

### Impact of induced pain on QOL

In this study, pain developed during the one-year follow-up in 57% of the patients and AI treatment was stopped because of pain in 9%. These results are similar to those reported in previous studies [2]. In a recent cross-sectional study of 68 women with breast cancer, Olufade et al. reported musculoskeletal pain in up to 64% of patients [32]. Patients suffering from AI-induced pain usually present with symmetric bilateral joint pain, most commonly affecting the wrists, hands and knees [33]. Our patients endorsed five different types of pain: upper or lower limb joint pain, diffuse pain, neuropathic pain, tendon pain and mixed pain [14]. Even with this relatively small sample, we found that the types of pain induced had significantly different impacts on QOL. Almost all types of pain were associated with subsequent changes in QOL. The magnitude of the effect also depended on the type of pain. For example, neuropathic pain had a strong negative association with future perspective and all pain types were associated with increased arm symptoms. Fenlon et al. also reported a negative association of pain with quality of life, based on scores for SF36 domains and FACT scores for QOL questionnaires [8, 34]. More recently, Olufade et al. showed, with the SF36 short form, that physical and mental QOL scores for breast cancer patients with pain were worse than those for breast cancer patients without pain [32]. This study’s results, showing poor QOL scores for patients with specific type of pain, are important because the associations of different types of pain with subsequent QOL have never before been considered. They demonstrate the importance of determining the type of AI-induced pain for specific diagnosis, so that the most appropriate type of management can be offered. Indeed, joint pain is not treated in the same way as diffuse or neuropathic pain. This clinical difference is of particular relevance for both patients and clinicians. Fenlon et al. reported a prevalence of 69% for stiffness or joint or muscle pain after primary cancer surgery (before adjuvant therapy), in 543 patients. However, this percentage decreased to 28% if the analysis was restricted to joint pain and stiffness [34].

Also of note in this context is the 9% of patients who had to stop AI treatment because of pain in this study. A five-year course of AI treatment is recommended, because it has been shown to increase disease-free survival and to decrease the frequency of contralateral breast cancer more effectively than adjuvant tamoxifen treatment [35–38]. However, adverse effects often result in poor compliance with AI treatment, with up to 50% of patients not taking AI as prescribed, and discontinuation rates of 20% within the first year of use [39]. AI-induced pain is the most common reason for poor compliance and the discontinuation of AI treatment [40, 41]. It is therefore important to inform patients and prescribers that pain is a common adverse effect of AI treatment. The potential benefits of treatments that could effectively reduce this pain on increasing treatment compliance should be examined. Even simply being sure to explain symptoms and describe risk factors for pain could potentially improve compliance with treatment. Guidelines are now required for general practitioners and oncologists.

### Study limitations

This study has several important limitations that must be taken into account when interpreting its findings. The key limitations include study design (non-controlled observational study) and the relatively small sample size (*n* = 135). This small sample size may have resulted in insufficient power to detect QOL predictors, for example. Additional studies with larger samples are required to confirm our results and develop and validate clinical tools that can be used accurately by oncologists in the context of AI treatment. Moreover, all our patients were Caucasian, which precluded racial differences to be evidenced. The use of analgesics was authorized and may have limited the prevalence of pain and its severity. Moreover, the women recruited did not have metastasis and, therefore, had a better prognosis. They might, therefore, have a better QOL than other women.

### Perspectives

Larger prospective studies are required to better understand and quantify AI-related pain and its consequences. Having breast cancer and receiving curative treatments for this disease are difficult experiences for patients, and may cause psychological distress. This distress and the poor quality of life associated with it may make it more likely that the patients will suffer AI-related pain. This study’s findings suggest that extensive patient assessment before the initiation of AI treatment would be useful. It would include osteoarticular features but also psychological dimensions and cancer-related quality of life, as proposed by Din [13]. Patient education, treatments that address distress, and/or treatments that effectively reduce pain might increase compliance with AI treatment. Oncologists could be encouraged to provide more appropriate, earlier treatment [42, 43]. Moreover, oncologists feel that the establishment of guidelines would improve their practices [13].

### Conclusion

Quality of life is affected very early in the course of breast cancer. Worse baseline functional and symptom domain scores were associated with a history of previous chemotherapy and taxane treatment, ganglion resection and psychological factors, such as harm avoidance, pain magnification (a catastrophizing domain) and psychological function (anxiety and depression). These predictors should not be underestimated and should be taken into account as soon as possible when initiating AI treatment.

Moreover, a poorer QOL score may increase the likelihood of pain being induced by aromatase inhibitor treatment. Indeed, AI-related pain is frequent and can develop in many patients who are initially pain free. AI-related pain is not limited to arthralgia, and can be classified into five pain syndromes. These different types of pain show different patterns of associations on subsequent QOL scores. A precise classification of pain syndromes and their risk factors and QOL impact should be provided to both oncologists and patients, to improve management and, potentially, compliance with AI treatment. Research to identify the treatments that effectively address the side effects of AI treatment (including pain) and evaluating the beneficial effects of these interventions on AI treatment compliance is warranted.
